# Comparing race, gender, age, and career categories in recognizing and grouping tasks

**DOI:** 10.7717/peerj.9156

**Published:** 2020-05-15

**Authors:** Jingjing Song, Lin Li

**Affiliations:** Institute of Applied Psychology, China University of Geosciences, Wuhan, P. R. China

**Keywords:** Multiple categories, Race, Gender, Category recognition task, Category recognition, Category preference

## Abstract

The purpose of our research was to compare how participants weighed age, gender, race, and career categories in recognizing and grouping tasks. In Study 1, we used a category recognition task to compare participants’ speeds in recognizing information from different categories. The results showed that participants recognized the gender information most quickly, followed by career, race, and age information. In Study 2, a categorization task was used to compare participants’ category preferences. The results showed that the career category had the greatest weight, and the gender category had the lowest weight. Two targets who had different career identities were more possible considered as belonging to different groups than two targets with different gender, race or age identities. Our results have implications in understanding the weight of different categories, with gender and career category are the most important category that affects perception and evaluation.

## Introduction

Simple categories (for example, gender) divide complex social memberships into different groups and can help people rapidly evaluate others. Since there are multiple simple categories, there are multiple group identities. There have been several studies related to the perception and evaluation of multiple-category targets (Kang et al., 2014; Neuberg & Sng, 2013; Remedios et al., 2011; Song et al., 2017; Wang et al., 2015). When perceiving and evaluating a multiple-category target, a whole set of categories (e.g., gender, age, and race) compete for attention (Freeman & Ambady, 2011) and may differ in weight (Weisman, Johnson & Shutts, 2015; Zhao & Bentin, 2008). The question remains of which category plays the most important role in the perception and evaluation of multi-category targets (Song & Zuo, 2016a).

In our study, we compared the weight of age, gender, race, and career categories across different tasks. We used two tasks to analyze which categories were most salient and easily recognized, and which category had greater preferences. Determining the question of which category is most fundamental could help in further understanding category encoding and the social perception of multiple-category targets. Moreover, our results can be valuable for creating effective and targeted intervention methods to reducing social biases by mainly focused on the fundamental category.

### Comparing the weight of simple categories with natural clues

Gender, race, and age categories have visible external features that can be identified at first glance (Fitousi & Wenger, 2013), and almost everyone can correctly and efficiently extract categorical information from targets’ faces. Moreover, people make inferential judgments based on these clues, and show category-based attitudes and behaviors toward the target (Baron & Banaji, 2006; Weisman, Johnson & Shutts, 2015). Thus, these three categories dominate the early stages of social perception (Fitousi, 2017).

Previous research on social perception and cognition has prioritized these three categories (Quinn & Macrae, 2005; Waxman, 2010; Weisman, Johnson & Shutts, 2015), particularly gender. Infants can distinguish faces by gender (Quinn et al., 2011), and preschool-age children show preference for children of their own gender. Gender is often an essential basis for children’s playmate selection, object and activity preferences, and perception and evaluation of others (Ruble, Martin & Berenbaum, 2006; Yee & Brown, 1994). Race is also a fundamental social category. While two and three-year-old children do not appear to share toys (Kinzler & Spelke, 2011) or choose friends (Shutts, Roben & Spelke, 2013) on the basis of race, five-year-old children have been shown to express explicit social preferences for individuals of their own race (Kinzler & Spelke, 2011). Age is also considered a fundamental social category guiding how we perceive others (Macrae & Bodenhausen, 2000).

However, these three categories do not carry equal weight in social perception. Many previous studies have compared the weight of the gender and race categories. One study showed that three-year-old children preferred friends based on gender, not racial, similarities between themselves and others (Shutts, Roben & Spelke, 2013). Another study demonstrated that when attempting to remember facts about individuals, participants were sensitive to gender but not race (Weisman, Johnson & Shutts, 2015). Zhao & Bentin (2008) also found that determining race is not the first step when processing faces. In general, gender might be a more fundamental social category than race (Weisman, Johnson & Shutts, 2015).

Some studies have also compared the weight of the gender and age categories. Evidence suggests that age often takes precedence over gender when perceiving targets (Bornstein, 1986; Kite, Deaux & Miele, 1991). People recognized age more quickly than gender (Zhao & Bentin, 2008), relied more on age than gender during sorting tasks (Bornstein, 1986), and made more age-stereotypical evaluations than gender-stereotypical evaluations of targets (Kite, Deaux & Miele, 1991). Additionally, research has shown how perceptions of sex and age interact. People pay less attention to the gender of an infant, but can quickly identify the gender category information of a young adult or old target (Cloutier, Freeman & Ambady, 2014). Therefore, the gender category has a greater weight in the perception of a young adult or old target, but carries less weight in the perception of an infant target.

Fewer studies have compared the weight of the race and age categories in social perception. Zhao & Bentin (2008) found that people recognized age more quickly than race. They also found interactions between the perceptions of race and age, with both Chinese and Caucasian participants classifying the age of Caucasian faces less accurately and more slowly than that of Chinese faces.

### Comparing the weight of simple categories with natural and social clues

The perception and evaluation of a target is substantially impacted by their social (occupation and wealth) and cultural identity (Song & Zuo, 2016a). Many previous studies have focused on wealth, with results showing that people, even children, prefer individuals of high socioeconomic status. Children aged 4–6 have been shown to prefer to befriend children with more wealth (Shutts et al., 2016) and give warmer evaluations of children with more resources (Li, Spitzer & Olson, 2014).

Career is also an important social category (Kunda, 1999; Zuo, 2016). Some occupations (e.g., doctors) are easily recognized by their uniforms, and different stereotypes are associated with different careers. For example, lawyers are stereotypically perceived as intelligent and argumentative, and librarians are stereotypically perceived as introverted and quiet (Kunda, 1999; Zuo, 2016). Additionally, many careers are considered to be gender-specific. Male-dominated occupations such as engineering and entrepreneurship are considered to require stereotypically male characteristics, while female-dominated occupations such as nursing and teaching are perceived to favor stereotypically feminine characteristics (Zuo, 2016). Thus, the career category plays a vital role in guiding social perceptions.

Few studies have compared the weight of categories with social clues and categories with natural clues. One exception is a study by Song & Zuo (2016a) who found that the functional significance of age was greater than wealth when evaluating warmth, while wealth played a more significant role than age when evaluating competence.

### The current study

Although previous studies have tried to compare the functional significance of different categories, these studies often only focused on comparing two categories. There has been no systematic comparison of the functional significance of multiple categories when perceiving and evaluating targets. Our study intended to simultaneously focus on four categories (age, gender, race, and career) that are important for social perception and can be easily recognized by external characteristics (face and upper-body clothes).

For the career category, we used a police officer and doctor for the following three reasons: (1) these two careers have universally recognized apparel (black police officer uniform and white doctor’s gown); (2) these two careers are considered male-dominated, which could reduce the interactions between career and gender on social perception (Zuo, 2016); and (3) these two careers have similar social economic statuses (SES) in China, which could decrease the influence of SES on social perception (Li, 2005).

Additionally, previous studies have mainly focused on Western children, not Chinese adolescents. It has been demonstrated that perception differs across participants because their identities, beliefs, and intentions influence which categories they use to identify others (Crisp & Hewstone, 2007; Turner & Reynolds, 2011). Thus, our study explored the functional significance of different categories among multiple Chinese adolescent participants.

Moreover, previous studies used different research tasks to compare the weights of multiple categories. Some studies focused on target perception, where researchers used an identity recognition task to compare the speed of age and gender recognition (Cloutier, Freeman & Ambady, 2014), or applied a memory confusion task to compare the weight of gender and race in social perception (Weisman, Johnson & Shutts, 2015), or used sorting tasks in which participants sorted images into groups (Yee & Brown, 1994). Other studies focused on social attitude and evaluation, where researchers used a social preference task to determine whether people chose friends based on gender or racial similarities (Shutts, Roben & Spelke, 2013), or used a stereotypical evaluation task to determine which category played a more important role when evaluating multiple-categories target (Song & Zuo, 2016a; Song & Zuo, 2016b). The sheer variety of different experimental tasks and categories in these studies makes comparison difficult.

When people encounter a target with multiple categories, they first recognize the target’s multiple social category information with all applicable categories activated in a parallel pattern, even though each category’s recognition speed is different. Next, a competition for mental dominance ensues, where the perceiver might unconsciously compare the weight of multiple categories (Crisp & Hewstone, 2007; Fitousi, 2017; Zuo et al., 2019). Finally, the perceiver socially infers and evaluates the target based on the dominant category. Thus, recognizing multiple categories, comparing the psychological meaning of each category, and extracting the dominant category are important parts of social perception, and influence later category-based attitudes and social evaluation. Focusing the social perception process, we chose a recognition task to compare category recognition speed on an unfamiliar target. We also used a categorizing preference task to determine which category played the most fundamental role in group classification.

The purpose of our research was to compare Chinese adolescents’ recognition speeds and categorizing preference of the age, gender, race, and career in the perception of a multiple-category target. We conducted two studies. In Study 1, a recognition task was used to compare the speed of identifying the age (young or old), gender (male or female), race (African or Asian descent) and career (police officer or doctor) of a multiple-category target. Based on previous studies, we assumed that participants would most quickly recognize the gender information, followed by age and then race. Since natural clue categories are more visible, easily identifiable, and repeatedly used to categorize people in daily life, they are more accessible to the perceiver (Song & Zuo, 2016a). Thus, we assumed that participants would recognize natural clue categories (gender, age, and race) more quickly than the career category.

In Study 2, we used a categorization task to identify preferences when perceiving multiple-category targets. We specifically wanted to explore that participants were more likely to place which pairs of targets (targets with different careers, genders, ages, or races) in a category. People cannot choose their age, gender, or race. However, since careers are self-selected, they may have higher functional and psychological significance than other categories when sorting strangers (Rozendal, 2003). Thus, we assumed there was a high possibility that the participants would sort people based on their career category, followed by gender, age, and race.

## Study 1: recognition speed when identifying simple categories

The purpose of Study 1 was to compare recognition speeds when identifying the race, age, gender, and career information of a multiple-category target. Photographs including the targets’ faces (providing age, sex, and race information) and upper-body clothing (providing career information) were presented to the participants. We then asked the participants to identify the multiple-category targets’ simple category information.

## Method

### Participants

Sixty-three students from a university in central China participated in this study. The participants’ ages ranged from 15 to 22 years (*M* = 18.21, *SD* = 0.94). There were 37 males (58.7%) and 26 females (41.3%). Thirty-one were from rural areas (49.2%) and 32 were from urban areas (50.8%). The required number of participants calculated using G*Power with Repeated Measures ANOVA (an alpha of 0.05, power at 80%, and an effect size f of 0.3) was 51 (Faul et al., 2009).

### Materials

There were 16 (2^4^) types of multiple-category target (e.g., old-female-Asian-police, young-male-African-doctor) photographs. The photograph subjects varied in age (old or young), gender (male or female), race (African or Asian), and career (police officer or doctor). We chose photographs from the Internet and slightly blurred them to minimize irrelevant face information. The targets’ facial expressions were neutral. We used computer-generated techniques to dress the targets in police officer or doctor uniforms. Before the experiment, twenty-nine college students determined whether the targets were male, female, of African descent, or of Asian descent by looking at their facial information (the options were yes or no). We only chose photographs for our studies that more than 90% of students rated typically male, female, of African descent, or of Asian descent. The twenty-nine college students also rated these photographs based on age, and we chose the photographs whose subjects’ ages ranged from 25 to 35 (typically young), and whose subjects’ ages ranged from 60 to 70 (typically old). Ultimately, we chose a total of 32 photographs for our study, with two different photographs for each target.

### Procedure

Our research conduct was consistent with recognized ethical guidelines. Permission was obtained from the ethics committees of the first author’s university (China University of Geosciences). All procedures performed involving human participants were in accordance with the ethical standards of the institutional research committee and with the 1964 Helsinki Declaration and its later amendments or comparable ethical standards. Participants gave written informed consent. They were given an overview of the study and were asked to recognize the targets’ category information (gender: male or female, race: African or Asian, age: old or young, and career: police officer or doctor) and press the corresponding keys on the keyboard as quickly and accurately as possible. When the study was complete, the participants were debriefed and thanked.

The experiments were controlled by a desktop computer with E-Prime. The photographs of the multiple-category target were presented one at a time in the middle of the screen. At the bottom of the screen, a pair of identity options was presented with a text description of each option randomly placed in either the left or right position (for example, “male” was on the left and “female” was on the right). The pair of options in the different trials included gender (male or female), age (old or young), career (police or doctor), and race (African or Asian).

The participants were asked to recognize the targets’ category information and press the correct corresponding option. Responses were made by pressing the left (“z”) or right (“m”) keys on the keyboard, and their response time was recorded. The participants were instructed to respond as quickly and accurately as possible.

Each trial in the experimental stage presented a target photo without a time limit. Once the participant responded by pressing the corresponding key on the keyboard, there was a blank screen for 500 ms, followed by the next target photograph. There was one block of 128 trials, based on the factorial combination of 32 photographs and four simple categories, with each photograph subject’s age, gender, race, and career requiring classification. The order of trials was randomly chosen by the computer.

### Results

All participants had an accuracy rate above 90%. Incorrect response times and extreme data points (beyond three standard deviations from the mean) were replaced with the means. We used SPSS19.0 for statistical analysis, and the category recognition response time means and standard deviations are shown in Table 1.

**Table 1:** The mean and standard deviation of the response time to the recognition of category information.

	Race information	Age information	Sex information	Career information
*M*	1480.04	1533.70	1298.39	1455.30
*SD*	354.11	342.56	331.49	328.19
*F*	46.99^∗∗∗^			

One-factor repeated measures ANOVA showed significant differences in the recognition time for race, age, gender, and career information (*F* (3,186) = 46.99, *p* < 0.001, ${\eta }_{\mathrm{p}}^{2}$ = 0.43). Post hoc tests showed that recognition speed for age (*M* = 1533.70, *SD* = 342.56) was significantly slower than race (*M* = 1480.04, *SD* = 354.11, *p* = 0.014, *d* = 0.15), career (*M* = 1455.30, *SD* = 328.19, *p* < 0.001, *d* = 0.23), and gender (*M* = 1298.39, *SD* = 331.49, *p* < 0.001, *d* = 0.70). There was no significant difference between the response times for race and career information (*p* = 0.24, *d* = 0.07). The response time for recognizing age, race, and career information was significantly slower than the gender information response time (*p* < 0.001, *d* = 0.70; *p* < 0.001, *d* = 0.53; *p* < 0.001, *d* = 0.48). Thus, we recognized the multiple-category target’s gender as the most quickly recognized category, followed by career, race, and age.

We further compared participants’ response times related to their recognition of different identity information within the same category (e.g., male and female for the gender category). The results of the pairwise *t*-test showed no significant difference between the response times for African (*M* = 1496.38, *SD* = 364.29) and Asian targets (*M* = 1463.71, *SD* = 379.64, *t*(62) = 1.14, *p* = 0.26, *d* = 0.09). However, the recognition response time for old targets (*M* = 1574.01, *SD* = 364.85) was significantly slower than for young targets (*M* = 1493.39, *SD* = 357.92, *t* (62) = 2.78, *p* = 0.07, *d* = 0.22), and the recognition response time for doctor targets (*M* = 1490.80, *SD* = 345.67) was significantly slower than for police officer targets (*M* = 1419.80, *SD* = 343.90, *t* (62) = 2.67, *p* = 0.01, *d* = 0.21). Finally, the recognition speed for female targets (*M* = 1323.94, *SD* = 341.89) was significantly slower than for male targets (*M* = 1272.85, *SD* = 354.57, *t* (62) = 1.90, *p* = 0.06, *d* = 0.15) (Table 2).

**Table 2:** The mean and standard deviation of the response time to the recognition of identity information.

	Race information		Age information		Sex information		Career information	
	African	Asian	Old	Young	Male	Female	Police	Doctor
*M*	1496.38	364.29	1574.01	1493.39	1272.85	1323.94	1419.80	1490.80
*SD*	1463.71	379.64	364.85	357.92	354.57	341.89	343.90	345.67
*F*	1.29		7.72^∗∗∗^		3.60^∗^		7.11^∗∗∗^	

We used 2 (gender of participants) × 2 (gender of targets) mixed-design ANOVA to analyze the effect of participant and target gender on recognition speed. Our results showed that the main effects of target and participant gender were not significant (*F* (1,61) = 2.23, *p* > 0.05, ${\eta }_{\mathrm{p}}^{2}$ = 0.04; *F* (1,61) = 0.95, *p* > 0.05, ${\eta }_{\mathrm{p}}^{2}$ = 0.02), but their interaction effect was significant (*F* (1,61) = 7.13, *p* < 0.01, ${\eta }_{\mathrm{p}}^{2}$ = 0.11). Simple effect analysis showed that the simple effect of target gender was not significant for female participants (*F* (1,61) = 0.59, *p* > 0.05, ${\eta }_{\mathrm{p}}^{2}$ = 0.01), but was significant for male participants (*F* (1,61) = 10.49, *p* < 0.01, ${\eta }_{\mathrm{p}}^{2}$ = 0.17). Male participants recognized male-category information faster ( *M* = 1278.20, *SD* = 337.17) than female-category information ( *M* = 1386.76, *SD* = 354.78).

### Study 2: Preferences in categorizing multiple- category targets

The purpose of Study 2 was to examine categorizing preferences when perceiving multiple-category targets. Participants were provided a photograph of a target with multiple categories and two other multiple-category test photographs that differed from the first target in only one category each. The participants were asked which of the two test photographs belonged to the same group with the first target photograph. For example, if participants used career information to guide their inferences about in-group and out-group membership, and the career category carried significant weight in the categorization task, then participants should have selected the member of the test pair that matched the target’s career and placed that test photograph in the same category as the target.

### Methods

### Participants

Sixty-three students from a university in central China participated in this study. The participants used in Study 2 were the same as in Study 1 and were randomly firstly asked to complete either Study 1 or Study 2. After completing the first task, the participants were then asked whether they would like to participate in another experiment (after a 10-minute rest). All subjects in our research were voluntarily involved in two studies.

### Materials

We used the same photographs from Study 1.

### Procedure

Our research conduct was consistent with recognized ethical guidelines. Permission was obtained from the ethics committees of the first author’s university (China University of Geosciences). Participants gave written informed consent. They were given an overview of the study and were asked to complete the tasks carefully. Finally, they were debriefed and thanked.

We used E-prime for our experiments. After the introduction, participants were able to see a combination of photographs in each trial, with the target photograph at the top and two test photographs at the bottom of the screen. Participants were asked which one of the two test photographs belonged to the same group with the target photograph, and to press the corresponding key on the keyboard. Responses were made by pressing the left (“z”) or right (“m”) keys on the keyboard. For example, if the participants thought the left test photograph belonged to the same group with the target photograph, they pressed the left “z” key. The experimenter did not give participants feedback on their responses, and the response times were recorded. The participants were instructed to respond as quickly as possible.

The multiple-category target photograph appeared at the top center of the computer screen. There were four types of targets in our study: (A) young-Asian-man-doctor, (B) old-Asian-man-doctor, (C) young-Asian-woman-doctor, and (D) old-Asian-woman-doctor (each type included two different photographs). Two multiple-category test photographs appeared simultaneously at the bottom of the computer screen (one at the bottom left and one at the bottom right). Each of these two test photographs differed from the target in only one category, but the other three categories were the same.

Take the young-Asian-man-doctor target (coded as A), for example. Four types of test photographs were presented at the bottom of the screen: old-Asian-man-doctor (different from the target in the age category, coded as A1), young-Asian-woman-doctor (different from the target in the gender category, coded as A2), young-Asian-man-police (different from the target in the career category, coded as A3), and young-African-man-doctor (different from the target in the race category, coded as A4). Since there were two test photographs for each trial, there were six combinations of photographs (A-A1A2, A-A1A3, A-A1A4, A-A2A3, A-A2A4, and A-A3A4).

There was one block of 96 experimental trials: 6 * 2 (balance the position of two test photographs) * 2 (each type of multiple category target included two photographs) * 4 (four types of targets presented in the top center of the screen). The trial order was randomly chosen by the computer. Each trial in the experimental stage first presented a combination of photographs without a time limit. Once the participants responded by pressing the corresponding key on the keyboard, there was a blank interval for 500 ms.

## Results

Since trends were consistent for the four types of targets in our analysis, we have chosen to present only the detailed results related to the young-Asian-man-doctor target (A). We first compared the weight of the gender and age categories. For target A, the two other test photos were of an old-Asian-man-doctor (A1) and a young-Asian-woman-doctor (A2). We used chi-square analysis to compare the actual frequency and the expected frequency of the categorization task, and the result was significant (*χ*^2^ = 10.73, *df* = 1, *p* < 0.001). Participants were likely to group A and the young-Asian-woman-doctor (A2) together (*N* = 152) more than A and the old-Asian-man-doctor (A1) (*N* = 100) (Table 3). The participants were more likely to combine two targets of different genders in one group than two targets with different ages. Therefore, the age category had a greater weight than gender in the categorizing task.

**Table 3:** The frequency of clarifying one of the two test photographs and the target photographs (young-Asian-men-doctor) as one group.

	A1,A2	A1,A3	A1,A4	A2,A3	A2,A4	A3,A4
A1	100	193	208			
A2	152			210	210	
A3		59		42		97
A4			44		42	155
sum	252	252	252	252	252	252

**Notes.**

A young-Asian-men-doctor A1 old-Asian-men-doctor A2 young-Asian-women-doctor A3 young-Asian-men-police A4 young-African-men-doctor

The number 100 in the table refer to that the frequency of clarify the A with A1 together as one group in the categorization task of clarify the target A with A1 and A2, and the number 152 refer to that the frequency of clarify the A with A2 together as one group. 2(balance the position of the test photographs) *2(each type of multiple-category target have two difference photographs)*63(the number of participants) = 252.

We then compared the weight of the career and age categories. The two other test photos were of an old-Asian-man-doctor (A1) and a young-Asian-man-police (A3). The chi-square analysis results were also significant (*χ*^2^ = 71.25, *df* = 1, *p* < 0.001). Participants were more likely to group A and the old-Asian-man-doctor (A1) together (*N* = 193) than A and the young-Asian-man-police (A3) (*N* = 59) (Table 3). Thus, the career category showed greater differentiating effects than the age category in the categorizing task. Participants were more likely to group two targets of different ages together than two targets with different careers.

We then compared the weight of the race and age categories. The test options were an old-Asian-man-doctor (A1) and a young-African-man-doctor (A4). The chi-square analysis results were significant (*χ*^2^=106.73, *df* = 1, *p* < 0.001). Participants were more likely to group A and the old-Asian- man-doctor (A1) together (*N* = 208) than A and the young-African-man-doctor (A4) (*N* = 44) (Table 3). They selected the member of the test pair who matched the target’s race more often than the target’s age. Thus, the functional significance of the race category was higher than the age category in the categorizing task.

Next, we compared the weight of the career and gender categories. The test options were a young-Asian-man-police (A3) and a young-Asian-woman-doctor (A2). The chi-square analysis results were significant (*χ*^2^=112.00, *df* = 1, *p* < 0.001). Participants were more likely to group A and the young-Asian-woman-doctor (A2) together (*N* = 210) than A and the young-Asian-man-police (A3) (*N* = 42) (Table 3). Thus, the functional significance of the career category was higher than the gender category.

We then compared the weight of the race and gender categories. The options were a young-African-man-doctor (A4) and a young-Asian-woman-doctor (A2). The chi-square analysis results were significant (*χ*^2^=112.00, *df* = 1, *p* < 0.001). Participants were more likely to group A and the young-Asian-woman-doctor (A2) together (*N* = 210) than A and the young-African-man-doctor (A3) (*N* = 42) (Table 3). Thus, race showed higher functional significance than gender.

Finally, we compared the weight of the career and race categories. The test options were a young-Asian-man-police (A3) and a young-African-man-doctor (A4). The chi-square analysis results were significant (*χ*^2^=13.35, *df* = 1, *p* < 0.001). Participants were more likely to group A with the young-African-man-doctor (A4) (*N* = 155) than A with the young-Asian-man-police (A3) (*N* = 97) (Table 3). Thus, the functional significance of the career category was higher than the race category.

## Discussion

The goal of our study was to compare the weight of age, gender, race, and career in the social perception of multiple-category target. Study 1 showed that the recognition speed of gender information was fastest, followed by career, race, and then age. Additionally, male participants recognized male information faster than female information. Study 2 found that in the categorizing task, the career category had the greatest weight, followed by race, age, and gender. When two targets had different career identities, they were considered as belonging to different groups. In summary, participants recognized gender information quickly, but tended to group together targets of different genders.

The weight of simple categories was different between the two different tasks. This suggests that results differ as motivations differ (Casper, Rothermund & Wentura, 2015). The category more closely related to the task is usually the dominant category, while the category less closely related to the task plays a smaller role (Bodenhausen, 2010; Song & Zuo, 2016a).

### The recognition speed of identifying simple categories

Chinese college students recognized young targets more quickly than old targets, and male college students identified male targets more quickly than female targets. These results were consistent with previous studies where participants recognized inner-group identity information more quickly than outer-group identity information (Crisp & Hewstone, 2007; Kinzler & Spelke, 2011). People interacted more with inner-group members and believed those group members were diverse and distinct, identifiable by face and upper-body clothing, in contrast to their perception of outer-group members who were considered to be indistinguishable and stereotypical. Thus, the perceiver’s identity can affect the perception and cognition about the multiple-category target (Crisp & Hewstone, 2007).

Consistent with our hypothesis, participants recognized gender information faster than the other categories in Study 1. The gender category was the most influential during social perception. The participants in the current study were young college students, and their social environments emphasize gender more than the other categories. Since Chinese college students mainly interact with same-age and same-race college students, they regularly rely on gender categories to organize their friends (Hilliard & Liben, 2010). During adolescence, building intimate relationships is a critical task and so this group may be more sensitive to gender information. Such practices lead to an emphasis on the gender category (Hilliard & Liben, 2010).

We found that it took longer to recognize age than race and career information. This result was inconsistent with our hypothesis. In the photographs we used, race was represented using skin color, and career was represented using white or black professional attire. Both categories had very distinct external characteristics that could be judged quickly even without observing detailed facial features. This could be an essential reason why race and career category information were recognized more quickly than age.

### Preferences in categorizing multiple-category targets

Consistent with our hypothesis, the career category seemed the most powerful, robust and potent category in the categorizing task. Individuals cannot choose their natural categories (age, gender, and race), but they can select their career. Previous studies have confirmed that self-selected categories such as career can reflect an individual’s intentions and motivations better than inherent categories, and can therefore play a more dominant role in the evaluation of a multiple-category target (Rozendal, 2003).

Race had the second greatest impact in the categorization task. Since Chinese college students typically live in a single-race environment and come from a single-culture background, African individuals are typically assigned to their outer-group. This may have caused race to be an essential factor in the categorization task.

Contradictory to our hypothesis, we found that the gender category’s weight was the lowest in the categorization task for young college students. This finding seems inconsistent with previous evidence that gender is a particularly strong guide to young children’s social preferences and reasoning (Shutts, Banaji & Spelke, 2010). People without finding their mates are considered as incomplete, and the mates (mainly referring to the heterosexual) are called “the other half” in China. Thus, people tend to group people with different gender. Moreover, it might be because young college students are looking to find a partner and are therefore more sensitive to gender information, and the recognition speed of the gender information was the fastest. Therefore, the results of study two were not contradictory with the results of previous research and study one.

### Limitations and implications

There were several limitations to our study. First, the police officer and doctor wore different uniforms to indicate their career categories, but the stark clothing differences may have exaggerated the weight of the career category. Second, our sample only included young Chinese college students. Future studies with participants from other racial and age groups are necessary for further understanding our results. Third, there are intersections among categories during social perception. Stereotypical evaluations of race often contain a gendered component. Black people tend to be associated with masculinity, while Asians tend to be associated with femininity (Schug, Alt & Klauer, 2015). Since we could not clearly distinguish the functional significance of each category, more effective methods in future studies are needed to compare the unique effects of each category and analyze their interactions. Fourth, the evaluator and contextual factors are important variables that influence the accessibility of simple categories (Crisp & Hewstone, 2007; Deaux, 2012; Kinzler, Shutts & Correll, 2010; Turner & Reynolds, 2011; Song & Zuo, 2016b). More empirical studies and analyses are needed.

Our research sheds light on Chinese adolescents’ category encoding of an unfamiliar target. Gender is an important category that affects perception and evaluation. The prioritization of the gender category exists across different cultures, indicating that it may be systemic. Our results also demonstrate the importance of the career category. Career, the social category that people choose on their own, has a greater psychological significance than natural clue categories when categorizing and evaluating others. Therefore, we need to focus on gender and career when attempting to reduce social bias. When identifying others, we encourage people to not only pay attention to a target’s gender and career, but also their personal qualities in order to ameliorate category-based attitude and judgments, and to reduce social bias.

##  Supplemental Information

10.7717/peerj.9156/supp-1 Data S1 Recognition speed in the identification of simple categories The participants were asked to identify the multiple-category target’s simple-category information (gender: male and female, race: African-descent and Asian-descent, age: old and young, and career: police and doctor). The response time and accuracy were recorded.

10.7717/peerj.9156/supp-2 Data S2 Preferences in categorizing the multiple-category target The number refer to the frequency of clarify the target photograph with test photograph together as one group in the categorization task.

10.7717/peerj.9156/supp-3 Data S3 Categorical data For the target young-Asian-descent-man-doctor (A), when the two test photos were old-Asian-descent-man-doctor (A1) and young-Asian-descent-woman-doctor (A2), there are 2 (balance the sequence of two test photographs) * 2 (each type of multiple category targets included two photographs)*4 (four types of targets presented in the top middle section of the screen)=16 trail. The frequency data was got with the sum of choosing A1/A2 in each trail.

## Additional Information and Declarations

### Data Availability

The following information was supplied regarding data availability: The raw measurements are available in the Supplemental Files.
