# Supplementary material for: Comparing race, gender, age, and career categories in recognizing and grouping tasks
Source: PeerJ. 2020 May 15;8:e9156. doi: 10.7717/peerj.9156 (PMC7233271; doi:10.7717/peerj.9156)
Supplement: Data S3 — For the target young-Asian-descent-man-doctor (A), when the two test photos were old-Asian-descent-man-doctor (A1) and young-Asian-descent-woman-doctor (A2), there are 2 (balance the sequence of two test photographs) * 2 (each type of multiple category targets included two photographs)*4 (four types of targets presented in the top middle section of the screen)=16 trail. The frequency data was got with the sum of choosing A1/A2 in each trail. [file peerj-08-9156-s003.doc]

the multiple-category target photograph appeared in the center of the top of the computer screen. There were four types of targets in our study: (a) young-Asian-descent-man-doctor, (b) old-Asian-descent-man-doctor, (c) young-Asian-descent-woman-doctor, and (d) old-Asian-descent-woman-doctor (each type included two different photographs). Meanwhile, two multiple-category test photographs appeared in the bottom of the computer screen (one at the bottom left and one at the bottom right); each of these two test photographs differed from the target only in one category and was the same in the other three categories.

Take the target young-Asian-descent-man-doctor (code as A) for example. Four types of test photographs would be presented in the bottom section of the screen: old-Asian-descent-man-doctor (different from the target in the age category, code as A1), young-Asian-descent-woman-doctor (different from the target in the gender category, code as A2), young-Asian-descent-man-police (different from the target in the career category, code as A3), and young-African-descent-man-doctor (different from the target in the race category, code as A4). As there were two test photographs each trail, thus, there would be six combinations of photographs options (A-A1A2, A-A1A3, A-A1A4, A-A2A3, A-A2A4, A-A3A4).

There was one block of 96 experimental trials: 6 * 2 (balance the sequence of two test photographs) * 2 (each type of multiple category targets included two photographs)*4 (four types of targets presented in the top middle section of the screen).

For the target young-Asian-descent-man-doctor (A), when the two test photos were old-Asian-descent-man-doctor (A1) and young-Asian-descent-woman-doctor (A2), there are 2 (balance the sequence of two test photographs) * 2 (each type of multiple category targets included two photographs)*4 (four types of targets presented in the top middle section of the screen)=16 trail. The frequency data was got with the sum of choosing A1/A2 in each trail.
